# Safety and efficacy of fluoroscopy-guided urethral catheterization in case of failed blind or cystoscopy-assisted urethral catheterization

**DOI:** 10.1038/s41598-024-60224-1

**Published:** 2024-04-24

**Authors:** Sang Woo Kim, In Chul Nam, Doo Ri Kim, Jeong Sub Lee, Jeong Jae Kim, Bong Su Kim, Guk Myung Choi, Sung Eun Park

**Affiliations:** 1 Department of Radiology, Jeju National University School of Medicine, Jeju National University Hospital, 15, Aran 13-gil, Jeju, 63241 Republic of Korea; 2 Department of Radiology, Gyeongsang National University School of Medicine, Gyeongsang National University Changwon Hospital, 11 Samjeongja-ro, Seongsan-gu, Changwon, 51472 Republic of Korea

**Keywords:** Health care, Medical research, Urology

## Abstract

This retrospective study evaluated the safety and efficacy of fluoroscopy-guided urethral catheterization in patients who failed blind or cystoscopy-assisted urethral catheterization. We utilized our institutional database between January 2011 and March 2023, and patients with failed blind or cystoscopy-assisted urethral catheterization and subsequent fluoroscopy-guided urethral catheterization were included. A 5-Fr catheter was inserted into the urethral orifice, and the retrograde urethrography (RGU) was acquired. Subsequently, the operator attempted to pass a hydrophilic guidewire to the urethra. If the guidewire and guiding catheter could be successfully passed into the bladder, but the urethral catheter failed pass due to urethral stricture, the operator determined either attempted again with a reduced catheter diameter or performed balloon dilation according to their preference. Finally, an appropriately sized urethral catheter was selected, and an endhole was created using an 18-gauge needle. The catheter was then inserted over the wire to position the tip in the bladder lumen and ballooned to secure it. We reviewed patients’ medical histories, the presence of hematuria, and RGU to determine urethral abnormalities. Procedure-related data were assessed. Study enrolled a total of 179 fluoroscopy-guided urethral catheterizations from 149 patients (all males; mean age, 73.3 ± 13.3 years). A total of 225 urethral strictures were confirmed in 141 patients, while eight patients had no strictures. Urethral rupture was confirmed in 62 patients, and hematuria occurred in 34 patients after blind or cystoscopy-assisted urethral catheterization failed. Technical and clinical success rates were 100%, and procedure-related complications were observed in four patients (2.2%). The mean time from request to urethral catheter insertion was 129.7 ± 127.8 min. The mean total fluoroscopy time was 3.5 ± 2.5 min and the mean total DAP was 25.4 ± 25.1 Gy cm^2^. Balloon dilation was performed in 77 patients. Total procedure time was 9.2 ± 7.6 min, and the mean procedure time without balloon dilation was 7.1 ± 5.7 min. Fluoroscopy-guided urethral catheterization is a safe and efficient alternative in patients where blind or cystoscopy-assisted urethral catheterization has failed or when cystoscopy-urethral catheterization cannot be performed.

## Introduction

Urethral catheterization is a medical procedure widely used in patients who require urinary drainage or urine collection for measurement or continuous hemodynamic monitoring in critical care units^1–3^. Urethral catheterization is conducted in various clinical contexts, including outpatient, inpatient, and emergency settings, and typically involves blind urethral catheterization for catheter insertion^4^. However, blind urethral catheterization may fail or be difficult in some cases because of various factors, such as anatomical variations, urethral stricture, or obstruction, leading to potential iatrogenic urethral injuries^4–6^. In a recent prospective study, Stefanie et al.^4^ reported an iatrogenic urethral injury rate of 6.2 ± 3.8 per 1000 catheterizations, confirming that iatrogenic urethral trauma is a recurrent medical error observed universally across institutions, healthcare systems, and countries. Furthermore, iatrogenic urethral injury can lead to the development of urethral stricture disease^7,8^, which can increase the failure rate of urethral catheterization, necessitating repeated attempts at catheter insertion and ultimately increasing the risk of recurrent urethral injury. Typically, if blind urethral catheterization fails, urethral catheterization through direct visualization with a cystoscope is recommended^9^. However, in various clinical settings, there can be instances where cystoscopy-assisted urethral catheterization fails or is difficult to attempt. In such situations, alternative methods, such as radiologic urethral catheterization, may be considered safer and more effective^10,11^. Radiologic urethral catheterization uses imaging techniques, such as fluoroscopy or ultrasonography, to guide catheter insertion into the bladder. This technique has shown potential advantages over blind catheterization, particularly in patients with challenging anatomies or in those who have previously failed blind urethral catheterization attempts^11–19^.

This study evaluated the safety and efficacy of fluoroscopy-guided urethral catheterization in patients who failed blind or cystoscopy-assisted urethral catheterization attempts as an alternative. Through a comprehensive review of existing literature, we discuss the potential benefits and limitations of fluoroscopy-guided catheterization and provide recommendations for its use in clinical practice.

## Materials and methods

The institutional review board of Jeju National University Hospital approved the study (JEJUNUH 2023-04-021). Owing to the retrospective nature of the study, the need for informed consent was waived. A retrospective review was conducted on our institutional database between 1 January 2011 and 1 March 2023, and patients were selected from the outpatient department and the inpatient department, including intensive care units, medical and surgical wards, and emergency departments. In general, when blind urethral catheterization failed, especially when performed by attending physicians or physicians from specialties other than urology, the patient would be referred to a urologist to either retry blind urethral catheterization or undergo cystoscopy-assisted urethral catheterization. If urethral catheterization still failed under the urologist's attempt or immediate intervention by the urologist was challenging, the patients were referred to an interventional radiologist to perform fluoroscopy-guided urethral catheterization. Additionally, during nights or weekends, fluoroscopy-guided urethral catheterization was primarily performed. Apart from fluoroscopy-guided urethral catheterization, suprapubic cystostomy to allow urethral resting or rigid cystoscopy-assisted urethral catheterization were also considered. The study included patients who had previous unsuccessful attempts at blind or cystoscopy-assisted urethral catheterization, subsequently underwent fluoroscopy-guided urethral catheterization. The exclusion criteria were defined as follows: (1) fluoroscopy-guided urethral catheterization without prior blind or cystoscopy-assisted urethral catheterization attempt; (2) routine catheter exchange for long-term use or blockage; (3) urethral injuries from trauma or surgery; (4) repeated fluoroscopy-guided urethral catheterization within 4 weeks; (5) suprapubic cystostomy; (6) absence of RGU evaluation.

We documented the patients’ medical histories and gathered data, including age, sex, and the presence of gross hematuria. Additionally, we reviewed the RGU in the picture archiving and communication system to evaluate urethral abnormalities, including urethral injury, urethral stricture, or anatomical variations. Urethral strictures were classified according to their anatomical location as follows: (1) meatal; (2) penile; (3) penobulbar; (4) bulbar; (5) bulbomembranous; (6) membranous; (7) prostatic; (8) bladder neck; (9) panurethral. Urethral injury was considered to have occurred if the following criteria were met:

Discontinuous urethra alignment or contrast leakage from the urethra on the RGU.

Or

Visible hematuria or presence of blood at the urethral meatus after multiple attempts of blind urethral catheterization.

We assessed the technical and clinical success rates, procedure-related complications, the time from request to urethral catheter insertion, fluoroscopy time, total radiation dose, presence of balloon dilation for urethral stricture, number of balloon dilation, duration of balloon dilation, total procedural time, and urethral injury rate. Technical success was defined as the successful passage of the guidewire into the urinary bladder, followed by the placement of a urethral catheter. Clinical success was defined as adequate urine drainage from the urinary bladder. Procedure-related complications were classified as adverse events occurring during catheterization, such as newly developed urethral injury or bleeding, after acquisition of the initial RGU.

### Fluoroscopy-guided urethral catheterization technique

All procedures were performed by one of four interventional radiologists with 3, 5, 11, and 19 years of experience in urological intervention. The procedures were performed in one of the two angio-suites (AlluraClarity FD20; Philips Healthcare and Artis zee ceiling; Siemens Healthcare).

First, a penile dressing was applied while the patient was supine, and urethral anesthesia was induced using a 2% lidocaine injection through a 5-Fr guiding catheter. A 5-Fr catheter was inserted into the urethral orifice, and the RGU was acquired to evaluate the urethral tract. Subsequently, the operator attempted to pass a 0.035-inch hydrophilic guidewire to the problematic segment of the urethra, leading to the urinary bladder. If the guidewire and guiding catheter could be successfully passed into the bladder, but the urethral catheter failed pass due to urethral stricture, the operator either attempted again with a reduced catheter diameter or performed balloon dilation according to their preference^20,21^. Finally, an appropriately sized urethral catheter was selected, and an endhole was created using an 18-gauge needle. The catheter was then inserted over the wire to position the tip in the bladder lumen and ballooned to secure it.

### Statistical analysis

Data analysis was performed using descriptive statistics, and the results are presented as mean ± standard deviation or percentages, as appropriate. Differences were considered statistically significant if the p-value was < 0.05.

All statistical analyses were performed using SPSS version 22 (IBM Corp, Armonk, NY, United States of America).

### Ethical approval

The Ethics Committee of Jeju National University Hospital approved this study and waived written informed consent because of the retrospective study design (approval number 2023-04-021). This study was conducted in accordance with the Declaration of Helsinki.

## Results

**Figure 1:**
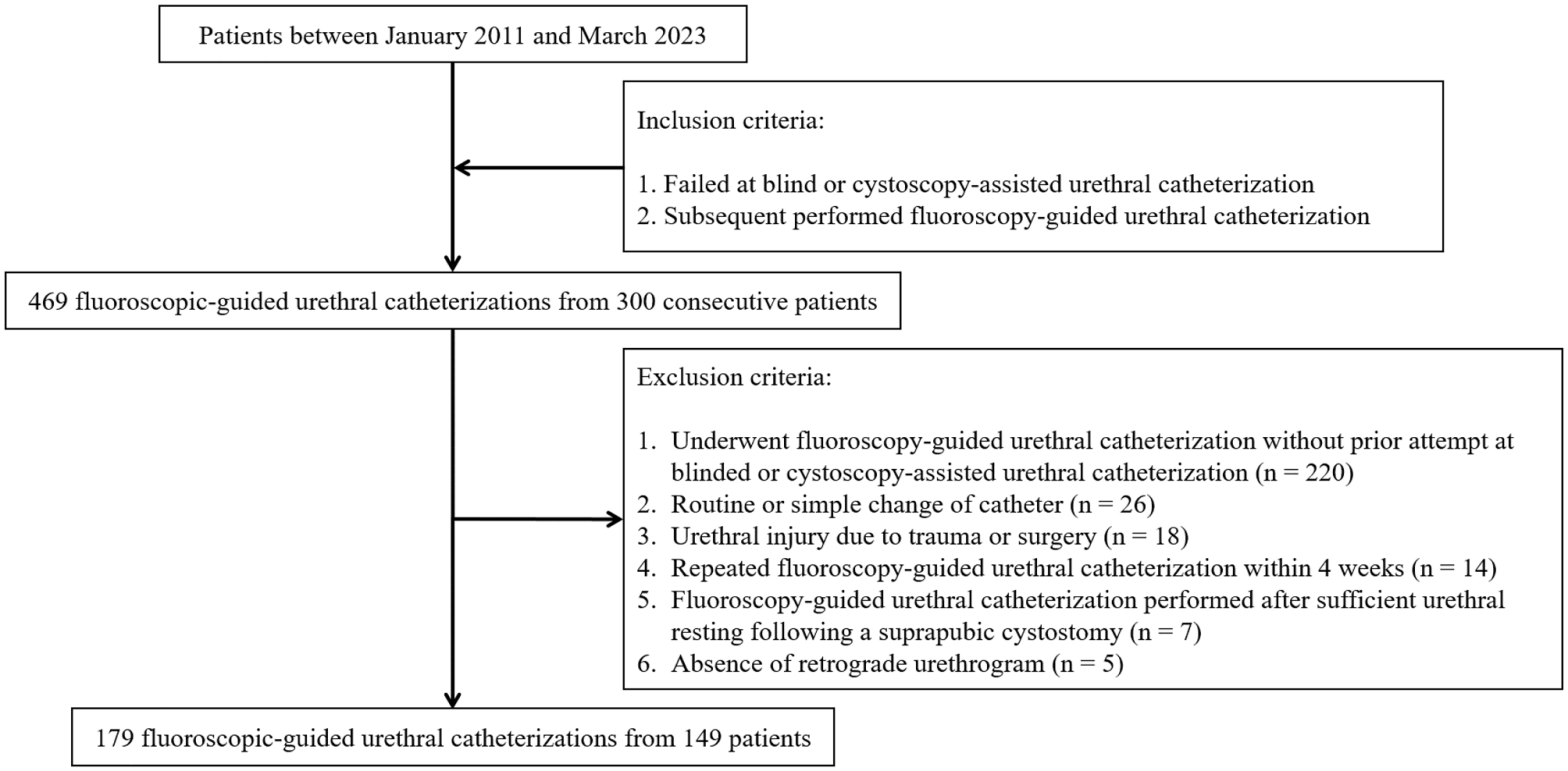
Flowchart of the case accrual process.

We initially identified 469 fluoroscopy-guided urethral catheterizations from 300 patients. Among them, a total of 290 fluoroscopy-guided urethral catheterizations from 151 patients were excluded. Finally, 179 fluoroscopy-guided urethral catheterizations from 149 patients were enrolled in this study. Figure 1 illustrates the accrual process. Patient demographics are summarized in Table 1.

**Table 1:** Demographics of the study population.

Age	73.3 ± 13.3 (range 22–94) years
Sex	100% male
Reason for urethral catheterization	
Urinary retention	65.9% (n = 118)
Urine output monitoring	12.3% (n = 22)
Hematuria	21.8% (n = 39)
Hematuria occurred after attempts of blind urethral catheterization	19% (n = 34)
Circumstances of urethral catheterization	
Emergency department	24.6% (n = 44)
Intensive care unit	12.8% (n = 23)
Medical or surgical ward	56.4% (n = 101)
Outpatient department	6.1% (n = 11)

**Figure 2:**
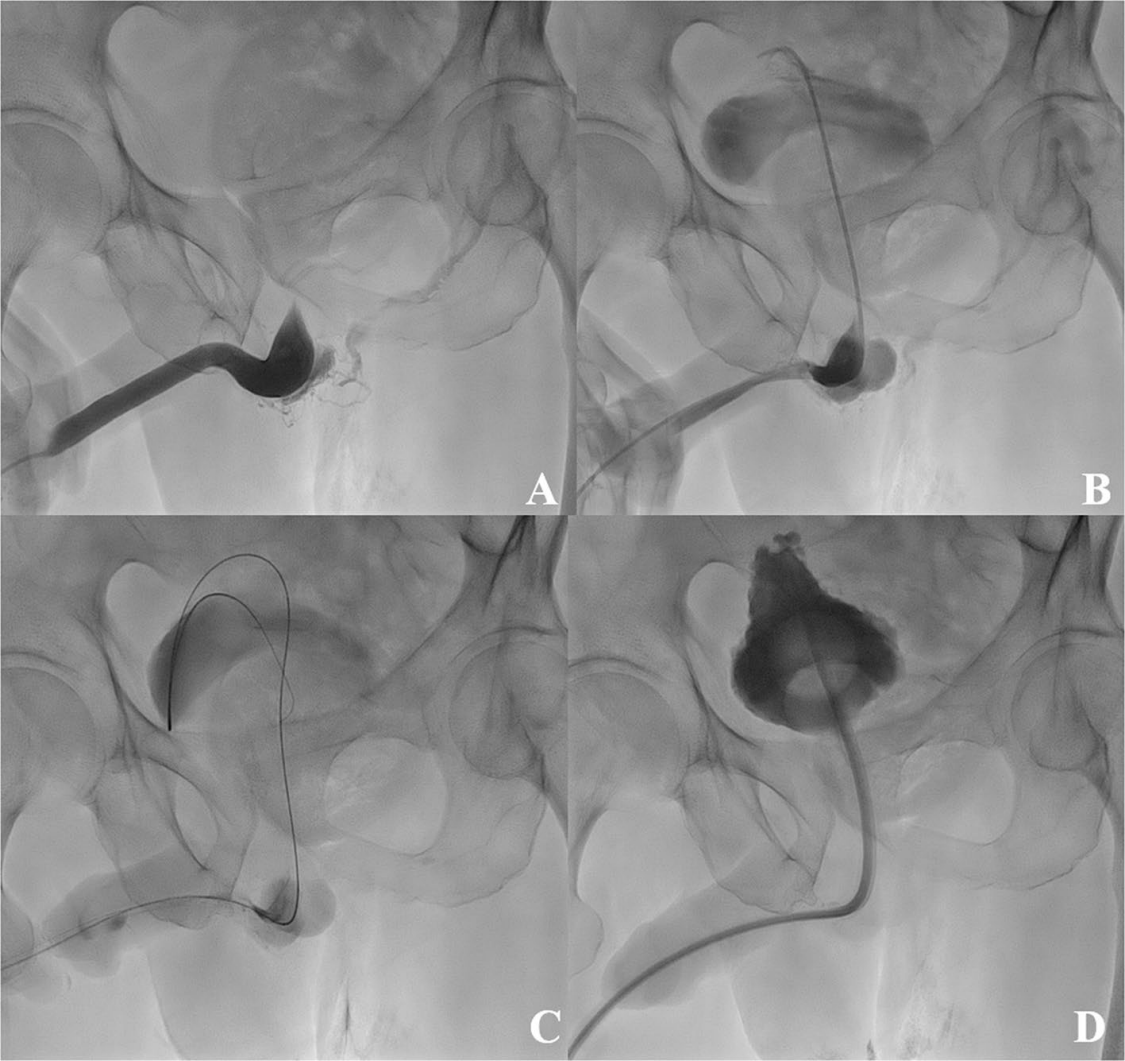
An 83-year-old male patient with severe urinary retention visited the emergency department, and blind urethral catheterization failed. Subsequently, he underwent fluoroscopy-guided urethral catheterization. (**A**) On retrograde urethrography, total occlusion of the prostatic urethra was observed, and contrast medium leakage into the venous structures around the bulbar urethra was noted. (**B**) Using a combination of a 5-Fr guiding catheter and a 0.035-inch hydrophilic guidewire, the injury and occluded segments were passed through, and a 5-Fr catheter was placed in the urinary bladder. Contrast was injected to confirm the catheter location in the urinary bladder. (**C**, **D**) Using the over-the-wire technique, a 20-Fr urethral catheter was placed in the urinary bladder, and the procedure was completed.

All patients underwent RGU at the beginning of the procedure to evaluate their urethral condition. Out of the study population, a significant stricture rate was identified, with 141 patients (94.6%) presenting with urethral strictures, highlighting the high prevalence of urethral stricture. Conversely, only eight patients (5.4%) showed no strictures. Among the urethral strictures, 120 (53.3%) had strictures in two or more locations. Urethral rupture was confirmed in 62 patients on RGU, and hematuria occurred in 34 patients after the failure of blind urethral catheterization. Of these, 23 had urethral rupture with hematuria after unsuccessful blind urethral catheterization, which was excluded because of an overlap between the two groups. Therefore, 73 patients (40.8%) experienced urethral injury during the attempted blind urethral catheterization. Figure 2 illustrates urethral strictures and injury after blind urethral catheterization. The RGU findings are summarized in Table 2.

**Table 2:** Findings of the retrograde urethrogram.

Fluoroscopy-guided urethral catheterization	100% (n = 179)
Urethral stricture (n = 225)	
Location	
Bladder neck	3.6% (n = 8)
Prostatic urethra	35.1% (n = 79)
Membranous urethra	10.7% (n = 24)
Bulbomembranous urethra	18.2% (n = 41)
Bulbar urethra	9.3% (n = 21)
Penobulbar urethra	12.9% (n = 29)
Penile urethra	3.6% (n = 8)
Urethra meatus	1.8% (n = 4)
Pan-urethra	4.9% (n = 11)
No urethral stricture	8
Urethral injury (n = 62)	
Location	
Bladder neck	1.6% (n = 1)
Prostatic urethra	8.1% (n = 5)
Membranous urethra	8.1% (n = 5)
Bulbomembranous urethra	53.2% (n = 33)
Bulbar urethra	16.1% (n = 10)
Penobulbar urethra	6.5% (n = 4)
Penile urethra	4.8% (n = 3)
Urethral meatus	1.6% (n = 1)

Technical and clinical success rates were 100%, and procedure-related complications were observed in four patients (2.2%). The mean time from request to urethral catheter insertion was 129.7 ± 127.8 min. The mean total fluoroscopy time was 3.5 ± 2.5 min. Concerning the X-ray dose assessment, the dose-area product (DAP) reported by the angiography machine was used. The mean total DAP was 25.4 ± 25.1 Gy cm^2^. Balloon dilation for urethral stricture was performed in 77 patients. The mean balloon diameter was 7.9 ± 0.3 mm, the mean number of balloon dilation was 2.1 ± 0.9, the mean time of balloon dilation was 2.4 ± 0.8 min, the mean total time of balloon dilation was 5.1 ± 3 min, the mean total procedure time was 9.2 ± 7.6 min, and the mean procedure time without balloon dilation was 7.1 ± 5.7 min. For patients in whom fluoroscopy-guided urethral catheterization was successful without balloon dilation, subsequent proper managements for urethral stricture (urethral dilation, visual internal urethrotomy, urethroplasty, etc.) were conducted by urologist^21^.

## Discussion

Blind urethral catheterization is the most commonly used method for inserting urinary catheters. However, this technique may sometimes fail, leading to potential complications, particularly in patients with urethral stricture disease, difficult anatomy, or a history of failed catheterization attempts. In such cases, fluoroscopy-guided urethral catheterization using the Seldinger technique^22^, which involves using a guidewire to obtain safe access to blood vessels, is suggested as a safer and more efficient alternative^11,13–19^.

In this study, we observed that the technical and clinical success rates were 100%, with only four cases (2%) of procedure-related complications. These results highlight the safety and efficacy of fluoroscopy-guided urethral catheterization in patients who experience difficulties with urethral catheter insertion or those with failed urethral catheterization attempts. In a similar study conducted by Kim et al.^11^, the success rate of urethral catheterization was 69.1%, which was significantly lower than that in the present study (*P* < 0.001). This may be because, in their study, only a hydrophilic guidewire was used to pass through the bladder after RGU, which can be technically challenging owing to inadequate support when passing through a stricture or injured segment. In our study, we used a 5-Fr guiding catheter with a J-shaped angled tip in addition to the guidewire to pass through the urethral stricture or injured segment stably and easily. Therefore, the guidewire passage was successful in all cases. In cases where the urethral catheter could not be inserted, balloon dilation was additionally performed, leading to a significantly higher overall success rate.

In our study, RGU revealed urethral injury in 62 patients (34.6%). The incidence of bulbomembranous urethral injury was significantly higher than that of other urethral segments. When combining injuries in the bulbomembranous, membranous, bulbar, and penobulbar urethra, injuries occurred in 83.9% of the cases. This is probably because of the anatomical features of the urethra, which is curved at approximately 90º at the penile-bulbar urethral junction and proximal bulbar urethra. When urethral stricture or urethral injury, such as urethral rupture or pseudo-tract, occurs in the bulbomembranous segment, attempting blind urethral catheterization may misalign the vector of force applied to the catheter in the direction of the urethra, causing the catheter to progress only to the site of the urethral injury, potentially worsening complications. This may explain why bulbomembranous urethral injury occurs at a higher rate than injury in other urethral segments. However, with fluoroscopy-guided urethral catheterization, the injury location and path of the urethral tract can be easily and accurately assessed using RGU. Using an appropriate guiding catheter with an angled tip and a hydrophilic guidewire, complications can be avoided while accurately identifying the urethral tract.

Compared to other studies, our study had a higher urethral injury rate (40.8%). Kim et al.^11^ reported definite urethral injury under RGU in 33 patients (24.3%). However, if we excluded the hematuria group and focused only on the 62 patients (34.4%) who showed definite urethral injury on RGU, there was no statistically significant difference compared with Kim et al.’s study, as seen in the chi-square test (*P* > 0.05). Gil et al.^23^ reported that gross hematuria in the urethral catheter was the most sensitive sign of urethral or urinary bladder injury in their cohort study and was often the only sign of such an injury. Therefore, the urethral injury rate reported by Kim et al. may have been underestimated because they excluded the urethral injury group that presented with hematuria. The combined values of the two groups, which included RGU-detected urethral injury and hematuria, may better reflect the actual urethral injury rate.

Hollingsworth et al.^24^ reported a 3.4% rate of urethral stricture erosion after short-term catheterization of less than 3 weeks. Kashefi et al.^6^ reported that the rate of traumatic urethral catheter insertion was 3.2 per 1000 inpatients. A recent prospective study in a multi-institutional setting across two national healthcare systems, the National Health Service in the UK and the Health Service Executive of Ireland, confirmed that the mean injury rate was 6.2 ± 3.8 per 1000 catheterization^4^. These differences in urethral injury rates may be due to a sampling bias. In contrast to other studies that included all patients who underwent urethral catheterization, our study focused on patients who experienced difficulties with catheterization or failed blind urethral catheterization, which introduced a sampling bias by overestimating the injury rate in the enrolled group and led to a higher urethral injury rate compared with other studies.

In our study, the mean total procedural time was 9.4 ± 7.7 min, and the mean procedural time without balloon urethroplasty was 7.2 ± 6 min. Given that the selected patient population comprised patients with failed blind urethral catheterization, which can be technically challenging and time-consuming, the mean procedural time in our study was considered acceptable. He et al.^25^ reported their clinical experience of wire-assisted urethral catheterization in difficult cases of male urethral catheterization. They evaluated the time spent during urinary catheterization, which was 15.4 ± 3.3 min in the wire-assisted urethral catheterization group. Based on a comparison with our study, the urethral catheter insertion time in their study was more than twice as long. This may have been due to the absence of fluoroscopic guidance and the lack of RGU, which could have resulted in more time-consuming procedures.

Difficult male urethral catheterization (DUC) is a common problem for the clinical physicians. Once a patients failed initial attempts at urethral catheterization, one of the following approaches can be used^9^; (1) passage of either a Glidewire, guide wire or filiform under direct vision (with the use of flexible or rigid cystoscopy); (2) blind passage of a filiform, guide wire, Glidewire or hydrophilic catheter followed by the advancement of a modified urethral catheter; (3) The Peel-away sheath placed on cystoscope/resectoscope technique; (4) The rigid ureteroscope placed inside the 22F Foley technique; (5) Suprapubic cystostomy; (6) The instillation of 60 cc of saline through the catheter. Direct visualization of the urethra enables identification of the source of resistance, obstruction, or other complication preventing blind urethral catheterization. Recent investigations^26^ have recommended the direct visualization in patients having high risk factors such as an enlarged prostate, urethral stricture, difficult insertions, false passages, and anticoagulation therapy. Lowe et al.^27^ discussed the management of the DUC by using a cystoscope in patients with trauma or post-operation states, yielding success rate was 85% (17/20). Beaghler et al.^28^ reported that flexible cystoscope-guided urethral catheterization is safe and effective in DUC patients with 96% of success rate (52/54). Blitz et al.^29^ reported cystoscope-guided urethral catheterization was used in 8 patients that had endoscopic prostate or urethral surgery in which catheters were placed with prior difficulty. With the cystoscope-guided urethral catheterization, they reported 100% of success rate (8/8). Through these previous studies, it is evident that in cases where blind urethral catheterization has failed, direct visualization via cystourethroscopy is recommended. However, due to the varying clinical settings of each hospital, cystoscopy-assisted urethral catheterization by a urologist may be difficult or impossible, or it may fail even if attempted on the initial try. In such cases, fluoroscopy-guided urethral catheterization, with its 100% of technical success rate and 2% of complication rate, can be utilized as an effective alternative.

In our study, the mean fluoroscopy time was 3.5 ± 2.5 min and the mean DAP was 25.4 ± 25.1 Gy cm^2^. Unfortunately, we could not find previously published data or references for comparison. However, we have considered the following measures to reduce concerns about radiation exposure and secure clinical benefits as follows: (1) Optimization and minimization efforts: We aimed to optimize the fluoroscopy technique and minimize radiation exposure by using pulsed fluoroscopy, limiting fluoroscopy time, and applying radiation shielding wherever possible; (2) Clinical necessity and benefits: In our study, 65.9% of patients required urethral catheterization due to acute urinary retention. In urgent situations where blind urethral catheterization failed, and cystoscopy by a urologist is impossible or difficult, fluoroscopy-guided urethral catheterization serves as a valuable alternative showing a 100% technical success rate and a very low complication rate, with a considerably short procedure time in our study; (3) Risk vs. Benefit analysis: Although urologists may be on standby for all situations, there could be instances where they are unavailable due to surgery or other reasons, posing a significant variable in urgent procedure scenarios. Moreover, if cystoscopy-assisted urethral catheterization fails, the next step is suprapubic cystostomy^21^. Suprapubic cystostomy, generally known to be safe and effective, is considerably more invasive since it requires piercing the lower abdominal wall to insert a large bore catheter into the bladder, compared to fluoroscopy-guided urethral catheterization. Additionally, suprapubic cystostomy can also entail the possibility of radiation exposure, depending on the operator and the procedure technique. Considering these points, if suprapubic cystostomy is being considered as the next step, the clinical benefit of performing fluoroscopy-guided urethral catheterization may outweigh the risk of radiation exposure.

Our study has some limitations, including its retrospective nature and the fact that it was conducted at a single center. Additionally, the sample size was relatively small, and the study population was highly selected, making it difficult to generalize the results to a broader population. Including the excluded 220 cases of fluoroscopy-guided urethral catheterization without a prior attempt of blind or cystoscopy-assisted urethral catheterization in our study could have allowed us to make a stronger assertion about the safety and efficacy of fluoroscopy-guided urethral catheterization. However, the 220 cases excluded were primarily those in which initial attempts at blind or cystoscopy-assisted urethral catheterization were difficult or failed, or urethral stricture was previously identified, suggesting future difficulty with blind or cystoscopy-assisted urethral catheterization. These cases were mostly managed with regular follow-ups at the urology outpatient department, scheduling elective fluoroscopy-guided urethral catheterization. Proceeding with the procedure on an elective schedule versus immediately performing fluoroscopy-guided urethral catheterization after a failed attempt at blind or cystoscopy-assisted urethral catheterization can result in entirely different conditions of the patient's urethra. In the latter case, the difficulty of urethral catheterization is anticipated to be higher. This is inferred from RGU findings of our study, where evidence of urethral injury was found in approximately 34.6%. Further studies with larger sample sizes and broader patient populations are required to confirm our findings. Second, fluoroscopy-guided urethral catheterization has the disadvantage that it can only be performed in facilities equipped with appropriate equipment for imaging guidance and requires trained personnel, which may not be available in certain medical settings. Finally, fluoroscopy-guided urethral catheterization can increase radiation exposure. Although we measured fluoroscopy time and radiation dose in our study, it is regrettable that the absence of other studies prevents comparison. Further research is necessary regarding the additional radiation exposure associated with fluoroscopy-guided urethral catheterization.

## Conclusion

Our study found that fluoroscopy-guided urethral catheterization is a safe and efficient alternative in patients where blind or cystoscopy-assisted urethral catheterization has failed or when cystoscopy-urethral catheterization cannot be performed. Early consideration of fluoroscopy-guided urethral catheterization in patients who are anticipated to fail or may have difficulty in blind urethral catheterization can significantly reduce the rate of iatrogenic urethral injury, which is associated with significant patient morbidity and financial burden.

## Data Availability

The datasets used and/or analysed during the current study available from the corresponding author on reasonable request.
